# Mobile health apps for cardiovascular risk assessment: a systematic review

**DOI:** 10.3389/fcvm.2024.1420274

**Published:** 2024-09-23

**Authors:** Fabian A. Chavez-Ecos, Rodrigo Chavez-Ecos, Carlos Vergara Sanchez, Miguel A. Chavez-Gutarra, Anandita Agarwala, Kiara Camacho-Caballero

**Affiliations:** ^1^ CHANGE Research Working Group, Carrera de Medicina Humana, Facultad de Ciencias de la Salud, Universidad Científica del Sur, Lima, Peru; ^2^ Red de Eficacia Clínica y Sanitaria, REDECS, Lima, Peru; ^3^ Department of Cardiovascular Disease, Mayo Clinic, Jacksonville, FL, United States; ^4^ Facultad de Medicina Humana, Universidad Nacional San Luis Gonzaga, Ica, Peru; ^5^ Center for Cardiovascular Disease Prevention, Baylor Scott and White Health Heart Hospital Baylor Plano, Plano, TX, United States

**Keywords:** systematic review, heart disease risk factors, mobile applications (apps), cardiology, risk assessment

## Abstract

**Introduction:**

mHealth apps (MHA) are emerging as promising tools for cardiovascular risk assessment, but few meet the standards required for clinical use. We aim to evaluate the quality and functionality of mHealth apps for cardiovascular risk assessment by healthcare professionals.

**Methods:**

We conducted a systematic review of MHA for cardiovascular risk assessment in the Apple Store, Play Store, and Microsoft Store until August 2023. Our eligibility criteria were based on the 2021 European Society Cardiology Guidelines on Cardiovascular Disease Prevention in Clinical Practice, the Framingham Risk Score, and the Atherosclerotic Cardiovascular Disease score. Our protocol was drafted using the Preferred Reporting items for Systematic Reviews and Meta-Analysis (PRISMA) guidelines. To assess quality, we used the validated Mobile Apps Rating Scale (MARS) score, which includes 19 items across four objective scales (engagement, functionality, aesthetics, and information quality) and one additional subjective scale. For functionality evaluation, we used the IMS Institute for Healthcare Informatics functionality scale. We performed data synthesis by generating descriptive statistics.

**Results:**

A total of 18 MHA were included in the review. The most common scores used were the Framingham score, ASCVD score, and Score 2. Only six apps achieved an overall score of 4 or greater in the MARS evaluation. The MHA with the highest MARS score was ESC CVD Risk Calculation (5 points), followed by ASCVD Risk Estimator Plus (4.9 points). In the IMS scale, four MHA had a high functionality score: ASCVD Risk Estimator Plus (5 points), ESC CVD Risk Calculation (5 points), MDCalc Medical Calculator (4 points), and Calculate by QsMD (4 points).

**Discussion:**

A gap exists in the availability of high-quality MHA designed for healthcare professionals to facilitate shared decision-making in cardiovascular risk assessment.

**Systematic Review Registration:**

The International Prospective Register of Systematic Reviews, identifier CRD42023453807.

## 1 Introduction

Cardiovascular disease (CVD) prevalence has been on the rise worldwide, with the number of individuals who have the disease increasing from 271 to 523 million from 1990 to 2019 (1). According to the World Health Organization (WHO), approximately 75% of cardiovascular diseases can be prevented, and reducing risk factors could significantly lower the burden of CVD (2). Since the first cohort of the Framingham study in 1948, early detection and treatment of cardiovascular risk factors have been proven useful in preventing myocardial infarction, stroke, and even death (3).

New technologies are emerging to assist in the diagnosis and treatment of cardiovascular diseases (4). One such technology is the Mobile Health applications (MHAs), as per the World Health Organization's definition “medical and public health practice supported by mobile devices, such as mobile phones, patient monitoring devices, personal digital assistants (PDAs), and other wireless devices.” (5) There are over 350,000 mobile health applications currently available on the market (6). MHAs have emerged as helping tools in decision-making for various diseases such as cardiovascular, endocrine, and psychiatric conditions (7–9). To what extent do MHAs meet the quality and functionality standards required in the evaluation of cardiovascular risk?

Although the number of applications has increased significantly nowadays and they play an important role in health, healthcare personnel who evaluate patients daily are particularly interested in applications that measure cardiovascular risk. Given the rapid growth in the number of such MHAs, how well do these tools align with the needs and expectations of healthcare professionals? Consequently, this systematic review aims to assess the quality and functionality of mobile health applications designed by healthcare professionals to measure cardiovascular risk.

## 2 Materials and methods

This study was performed according to the recommendations of the Preferred Reporting Items for Systematic Reviews and Meta-Analyses (PRISMA) (10) and we conducted an approach according to Gasteiger et al. methodology (11). The study protocol was registered in the International Prospective Register of Systematic Reviews (CRD42023453807). Our clinical question of interest is according to acronyms TECH (11), “What is the functionality and quality of mobile health applications that measure cardiovascular risk used by healthcare personnel?”

### 2.1 Eligibility criteria

We used the Clinical Guide Practical “2021 ESC Guidelines on cardiovascular disease prevention in clinical practice” (12), Framingham risk score (3), and ASCVD score considering the main outcomes in patients with comorbidities: (1) age; (2) chronic kidney disease (CKD); (3) familial hypercholesterolemia; (4) Diabetes Mellitus type 2 (DM2). In patients without comorbidities, we included the variables of the SCORE2 and SCORE2-OP: (1) age; (2) sex; (3) systolic blood pressure; (4) total cholesterol; (5) HDL-cholesterol; (6) LDL-cholesterol; (7) current smoker (13, 14).

### 2.2 Information sources and search strategy

We performed a search strategy in the Apple Store, Google Play Store, and Microsoft Store until August 2023. Additionally, we include a strategy search in PubMed and Scopus for identifying articles of our interest where MHA were included for validation. We included terms in Spanish, English, and Portuguese with the continuous terms “score” OR “risk”, AND “cardiovascular” (Supplementary Table S1).

### 2.3 Study selection

Duplicate MHAs were removed by two authors (FC & RC). After that, the MHAs were selected to identify potentially relevant characteristics according to the inclusion criteria; potential MHAs were evaluated and downloaded to assess their eligibility in the IOS and Android platforms. For paid applications, the authors purchased them to evaluate. Two researchers (FC & RC) assessed rigorously to exclude apps. Subsequently, the same authors evaluated the potential MHAs using the eligibility criteria to determine their inclusion. In case of disagreements, a third researcher (MC) made the final decision.

### 2.4 Data collection process

Two authors (FC & RC) independently extracted the following data from each included app using a standardized Microsoft Excel 2016 sheet form. This form contains the app name, platform, language, last update, developer, version, cost, cost-upgrade, privacy policy, size, recommendations based on Clinical Practice Guidelines (CPGs), adds, and type of score.

### 2.5 Quality evaluation

For quality evaluation, three reviewers (FC, RC & MC) assessed using the Mobile App Rating Scale (MARS), which comprises 19 items across four objective scales (engagement, functionality, aesthetics, and information quality) and an additional 4 items for the subjective quality scale. Each item is rated on a 5-point Likert scale: (1) inadequate, (2) poor, (3) acceptable, (4) good and (5) excellent (15). This tool was validated and suitable for the quality assessment (16).

### 2.6 Functionality evaluation

Three reviewers (FC, RC & MC) assessed functionality using the IMS Institute for Healthcare Informatics functionality scoring criteria. This score has 7 functionality criteria and 4 functional subcategories. If a function was present, it was coded as 1; otherwise, it was coded as 0. Functionality scores ranging from 0 to 11 were generated for each app. However, we have reached a consensus among the authors not to include the following items (evaluate data, intervene, remind or alert, and communicate) as they measure the patient's use of the application, which does not apply to our work aimed at health personnel. In the evaluation, we reached a consensus between the three reviewers in case of disagreements (17). We considered a MHA with high functionality (≥4 points), and low functionality (<4 points).

### 2.7 Statistical analysis

Data synthesis was performed by generating descriptive statistics (sums, mean, standard deviations, and percentages) on relevant items or combining these with forms of qualitative synthesis. We identified the highest scores of MHAs regarding quality and functionality; and presented these with a written description of their main features. Additionally, we used the intraclass correlation coefficient (ICC) for calculating interrater reliability for the ordinal MARS score. For figures, we used Python with Matplotlib to create the bar graphs for our data. Matplotlib helped us design and customize the graphs, while NumPy assisted with data calculations. This approach allowed us to clearly present the scores and other metrics.

## 3 Results

### 3.1 Mobile health apps selection

**Figure 1:**
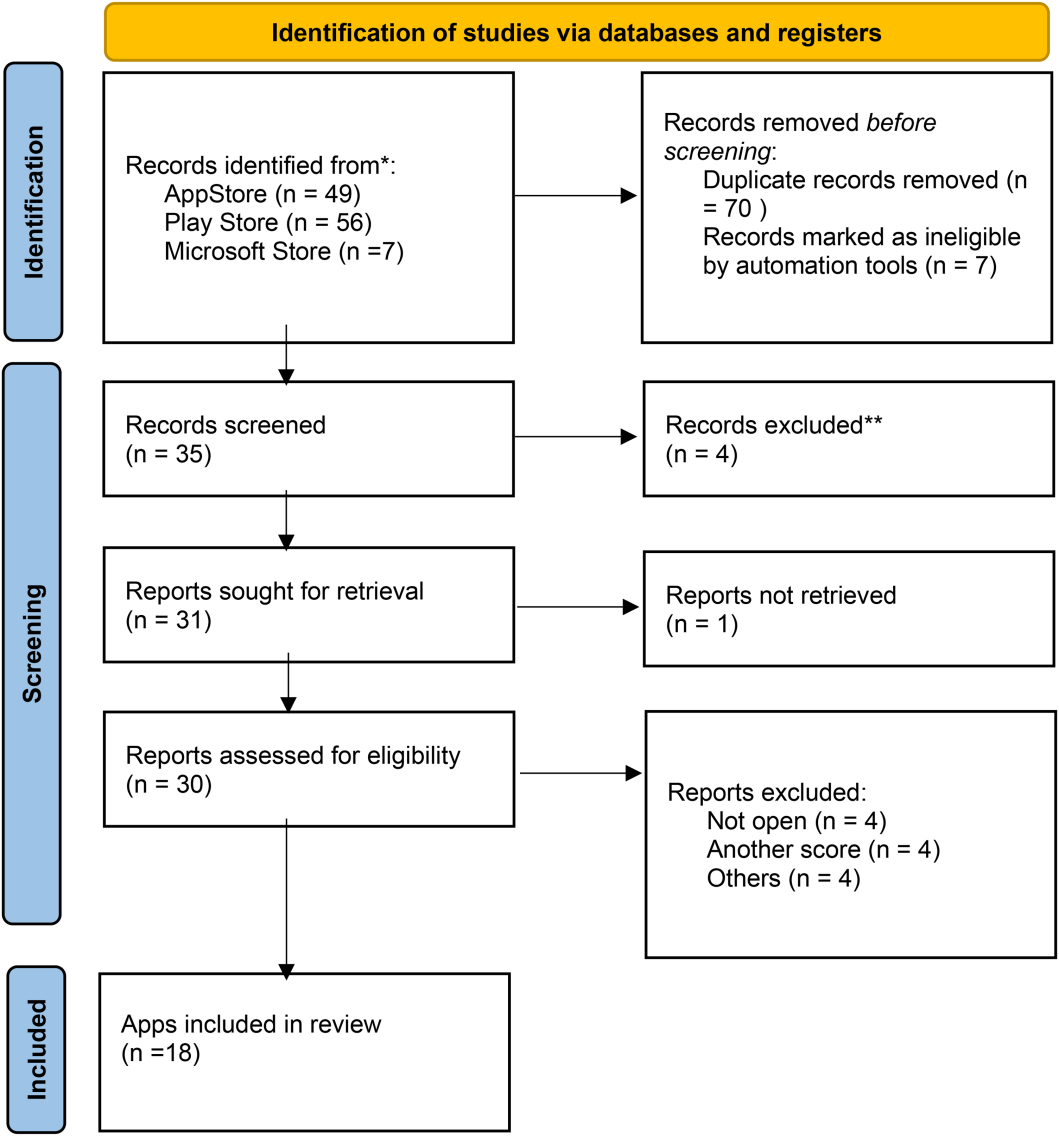
Flowchart of the mHealth apps selection process. *Consider, if feasible to do so, reporting the number of records identified from each database or register searched (rather than the total number across all databases/registers). **If automation tools were used, indicate how many records were excluded by a human and how many were excluded by automation tools.

A flow diagram describing our literature search process is provided in Figure 1. A total of 112 MHA were identified through the search strategy. Of these, 35 remained after removing duplicates and were evaluated. Twenty-nine were included in the assessment for eligibility and 12 MHA were excluded (Supplementary Table S1). Finally, we included 18 MHA for the MARS and IMS Institute for Healthcare Informatics score evaluation.

### 3.2 Characteristics of mobile health apps included

The characteristics of the MHAs included are presented in Table 1. 18 MHAs were included, and 11 of these are included in both IOS and Android platforms. The main language found is English in 16 apps; 5 apps offered Spanish language; apps with multiple languages were 4. Fifteen apps were free of cost and 16 apps had privacy policies. The mean size was 22.3 megabytes (IQR: 1.0–66.9). The MHA ESC CVD Risk Calculation included three scores (Framingham score, ASCVD score, and Score 2), being the Framingham score the most used and Score 2 the least used. CV Risk Estimation, MDCalc Medical Calculator, MedCalX, and Calculate by QxMD included two scores in their MHA. Eight MHAs included recommendations based on Clinical Practice Guidelines, the MHA ASCVD Risk Estimator Plus included (2019 ACC/AHA Guideline Primary Prevention of Cardiovascular Disease), ESC CVD Risk Calculation included (2021 ESC Guidelines on Cardiovascular Disease Prevention in Clinical Practice), CardioRisk Calculator included (2021 Canadian Cardiovascular Society Guidelines for the Management of Dyslipidemia for the Prevention of Cardiovascular Disease in Adults), and CV Risk Estimation, MDCalc Medical Calculator, MedCalX, Calculate by QxMD and Clinical Calculator PLUS included (2013 ACC/AHA Guideline on the Assessment of Cardiovascular Risk).

**Table 1:** Characteristics of the mHealth apps.

App name	Platform	Language	Last update	Developer	Cost	Cost-upgrade version	Privacy policy	Size	Adds	Recommendations based on CPG	Framingham score	ASCVD score	Score 2	IMS score
ASCVD Risk Estimator Plus	Android/IOS	English	25/7/2023	American College of Cardiology	Free	–	No	19 MB	No	2019 ACC/AHA Guideline on the Primary Prevention of Cardiovascular Disease	–	X	–	5
ESC CVD Risk Calculation	Android/IOS	English	3/5/2022	European Society of Cardiology	Free	–	Yes	20.5 MB	No	2021 ESC Guidelines on cardiovascular disease prevention in clinical practice	–	X	X	5
CardioCal	Android/IOS	English	2/3/2022	Pan American Health Organization	Free	Yes	Yes	51.3 MB	No	No.	–	–	–	3
Framingham Calc—Heart Age	Android/IOS	English	11/5/2023	Daniel Correia	Free	Yes	Yes	61.1 MB	Yes	No	X	–	–	1
CardioRisk Calculator	Android/IOS	English	31/5/2023	The University of British Columbia	Free	–	Yes	1.4 MB	No	2021 Canadian Cardiovascular Society Guidelines for the Management of Dyslipidemia for the Prevention of Cardiovascular Disease in Adults	X	–	–	1
CardioRiesgo Framingham	Android	Spanish	29/4/2023	Gemanepa	Free	–	Yes	3.61 MB	No	NR	X	–	–	3
Cardiovascular Risk Calculator	IOS	English	2018	Machealth Pty Ltd	Free	–	No	6.3 MB	No	No	X	–	–	2
CV Risk Calc	IOS	English	2022	Gangadhar Goud	Free	–	Yes	4.9 MB	Yes	No	–	X	–	1
CV Risk Estimation	IOS	English	2023	United Health Services, Inc	Free	–	Yes	1.5 MB	No	2013 ACC/AHA Guideline on the Assessment of Cardiovascular Risk	X	X	–	2
RapidASCVD: ASCVD Risk Calc	IOS/Android	English	NR	ClinCalc LLC	$0.99	–	Yes	3.6 MB	No	NR	–	X	–	2
Healhty Heart 2021	IOS	English	2021	Med Apps, LLC	$0.99	–	Yes	0.98 MB	No	NR	X	–	–	1
MDCalc Medical Calculator	IOS/Android	English	4/8/2023	MD Aware LLC	Free	–	Yes	44.8 MB	No	2013 ACC/AHA Guideline on the Assessment of Cardiovascular Risk	X	X	–	4
Regicor	IOS/Android	English Spanish Catalan	2022	Regicor	Free	–	Yes	27.1 MB	No	No	X	–	–	2
Calculadora de Riesgo Cardiova	Android	Spanish	17/05/2022	Sergio Garzon Hernandez	Free	–	Yes	2.84 MB	Yes		X	–	–	3
MedCalX	IOS	Spanish Chinese French Portuguese German	2020	Ossus GmbH	Free	Yes	Yes	40 MB	No	2013 ACC/AHA Guideline on the Assessment of Cardiovascular Risk	X	X	–	2
Cardio4ALL	IOS/Android	Portugues German French English Italian Dutch	01/02/2023	Phormula Group, Lda	Free	–	Yes	44.8 MB	No	No	–	–	X	2
Calculate by QxMD	IOS/Android	Spanish German French English Italian Portuguese	01/10/2022	WebMD Health Corporation	Free	–	Yes	66.9 MB	No	2013 ACC/AHA Guideline on the Assessment of Cardiovascular Risk	X	X	–	4
Clinical Calculator PLUS	IOS/Android	English	2022	Skyscape Medpresso Ins	$6.99	–	Yes	21.8 MB	No	2013 ACC/AHA Guideline on the Assessment of Cardiovascular Risk	X	X	–	3

CPG, clinical practice guidelines; NR, not reported.

### 3.3 MARS and IMS institute for healthcare informatics evaluation

**Figure 2:**
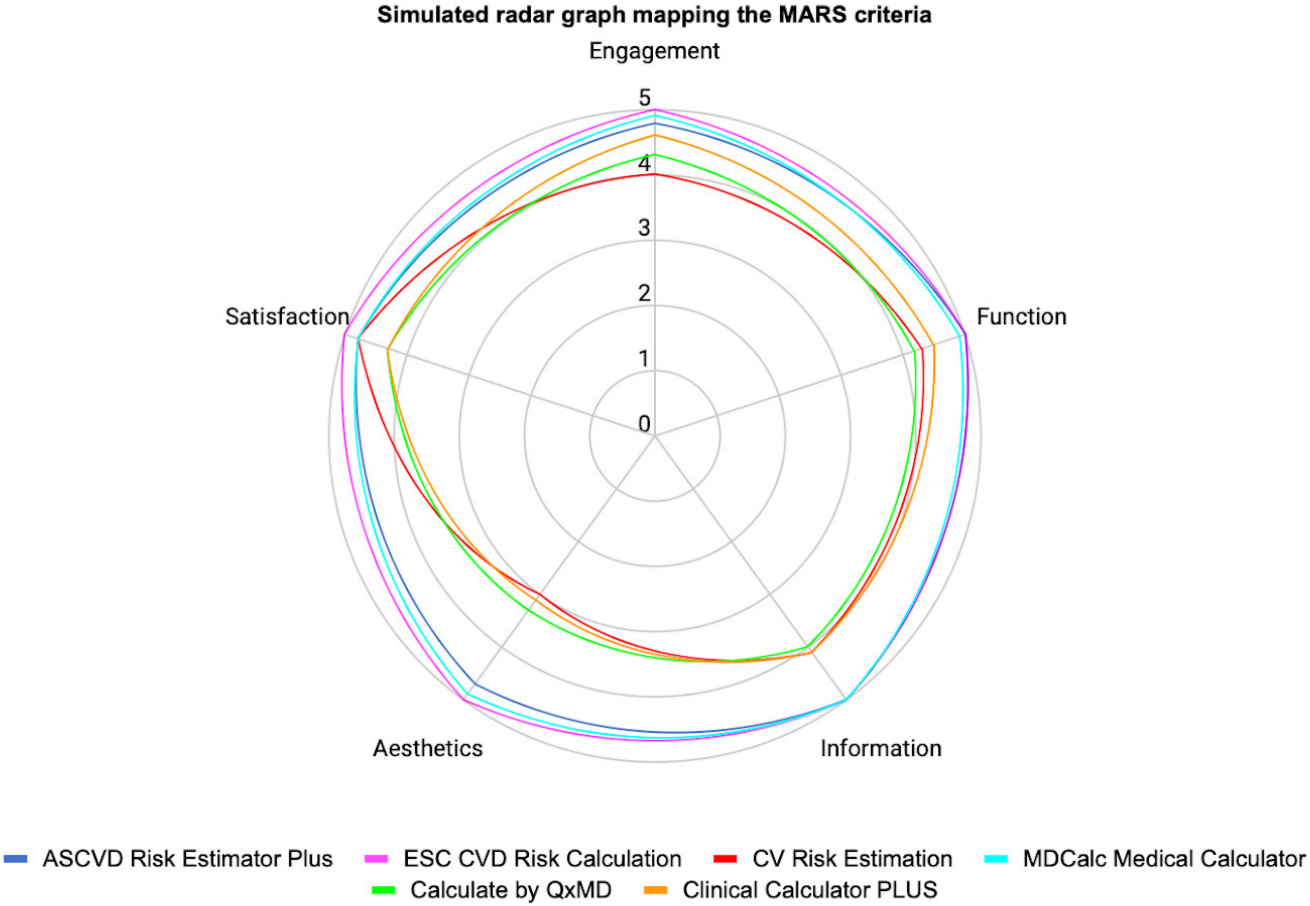
Simulated radar graph mapping the MARS criteria engagement.

**Figure 3:**
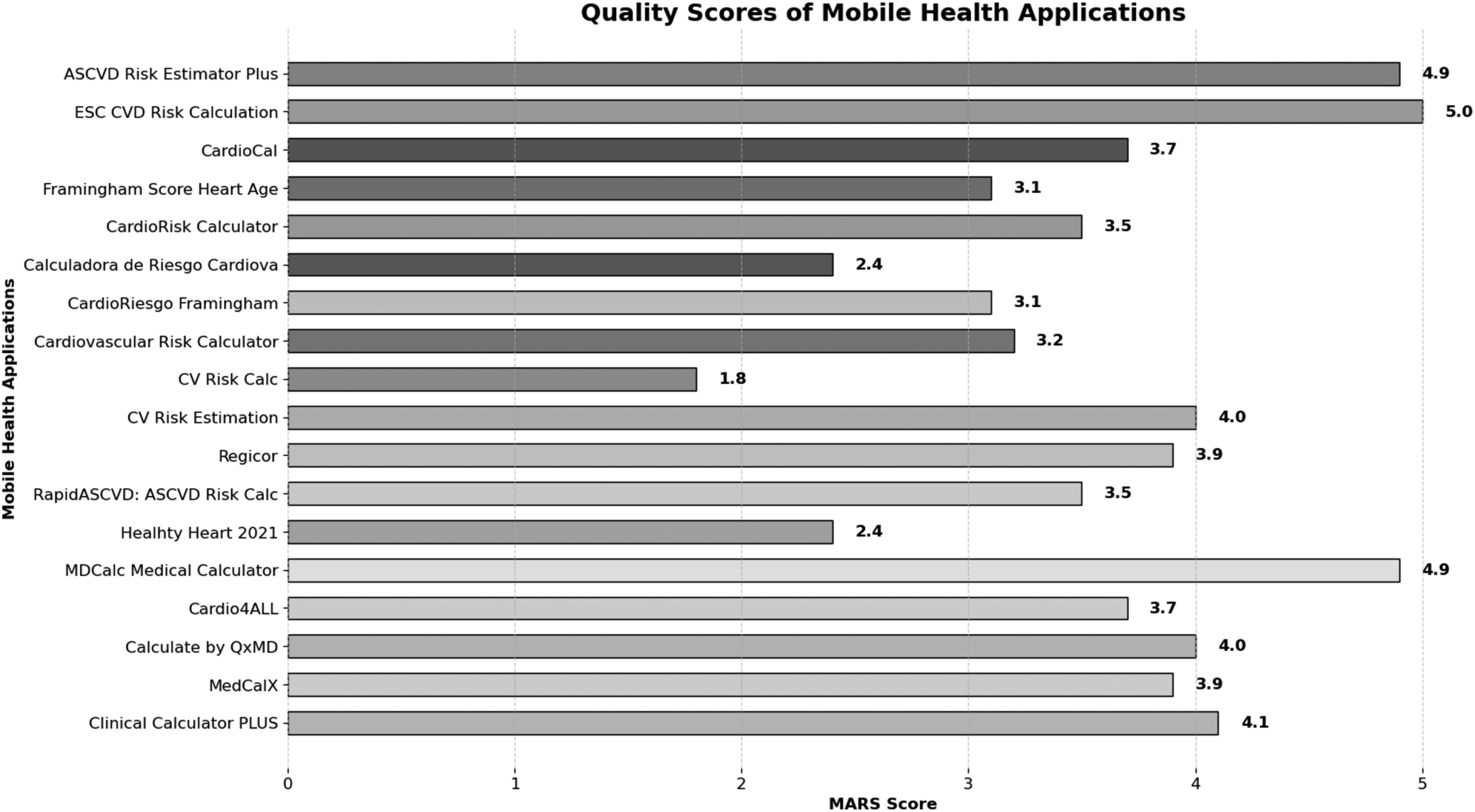
Quality scores of mobile health applications.

In MARS evaluation the MHA with the highest score was ESC CVD Risk Calculation (Engagement: 5.0, Function: 5.0, Information: 5.0, Aesthetics: 5.0, Satisfaction: 5.0, and Overall:5.0), the next were ASCVD Risk Estimator Plus (Engagement: 4.8, Function: 5.0, Information: 5.0, Aesthetics: 4.7, Satisfaction: 4.8, and Overall:4.9) and MDCalc Medical Calculator (Engagement: 4.9, Function: 4.9, Information: 5.0, Aesthetics: 4.9, Satisfaction: 4.8, and Overall:4.9). Three independent reviewers assessed the interrater reliability of four MHAs in a randomized manner (ICC = 0.91, CI 95% 0.83–0.96) (Table 2, Figures 2, 3).

**Table 2:** MARS evaluation of the mHealth apps included.

Name	Engagement	Function	Information	Aesthetics	Satisfaction	Overall
ASCVD Risk Estimator Plus	4.8	5.0	5.0	4.7	4.8	4.9
ESC CVD Risk Calculation	5.0	5.0	5.0	5.0	5.0	5.0
CardioCal	3.3	4.0	4.0	3.9	3.1	3.7
Framingham Score Heart Age	2.9	3.6	3.3	2.8	2.7	3.1
CardioRisk Calculator	3.2	4.1	3.6	3.7	3.1	3.5
Calculadora de Riesgo Cardiova	2.8	2.3	2.3	2.7	1.8	2.4
CardioRiesgo Framingham	3.2	4.0	3.1	3.2	2	3.1
Cardiovascular Risk Calculator	3.6	3.9	3.2	3.3	1.8	3.2
CV Risk Calc	1.5	2.0	1.7	3.0	1.0	1.8
CV Risk Estimation	4.0	4.3	4.1	3.0	4.8	4.0
Regicor	4.1	4.3	4.3	3.2	3.8	3.9
RapidASCVD: ASCVD Risk Calc	3.4	4.0	3.4	3.3	3.3	3.5
Healhty Heart 2021	2.9	3.0	2.1	2.0	1.8	2.4
MDCalc Medical Calculator	4.9	4.9	5.0	4.9	4.8	4.9
Cardio4ALL	4.2	4.3	2.7	3.3	3.8	3.7
Calculate by QxMD	4.3	4.2	4.0	3.3	4.3	4.0
MedCalX	4.4	4.0	4.0	3.1	3.8	3.9
Clinical Calculator PLUS	4.6	4.5	4.1	3.1	4.3	4.1

(1) inadequate, (2) poor, (3) acceptable, (4) good and (5) excellent.

**Figure 4:**
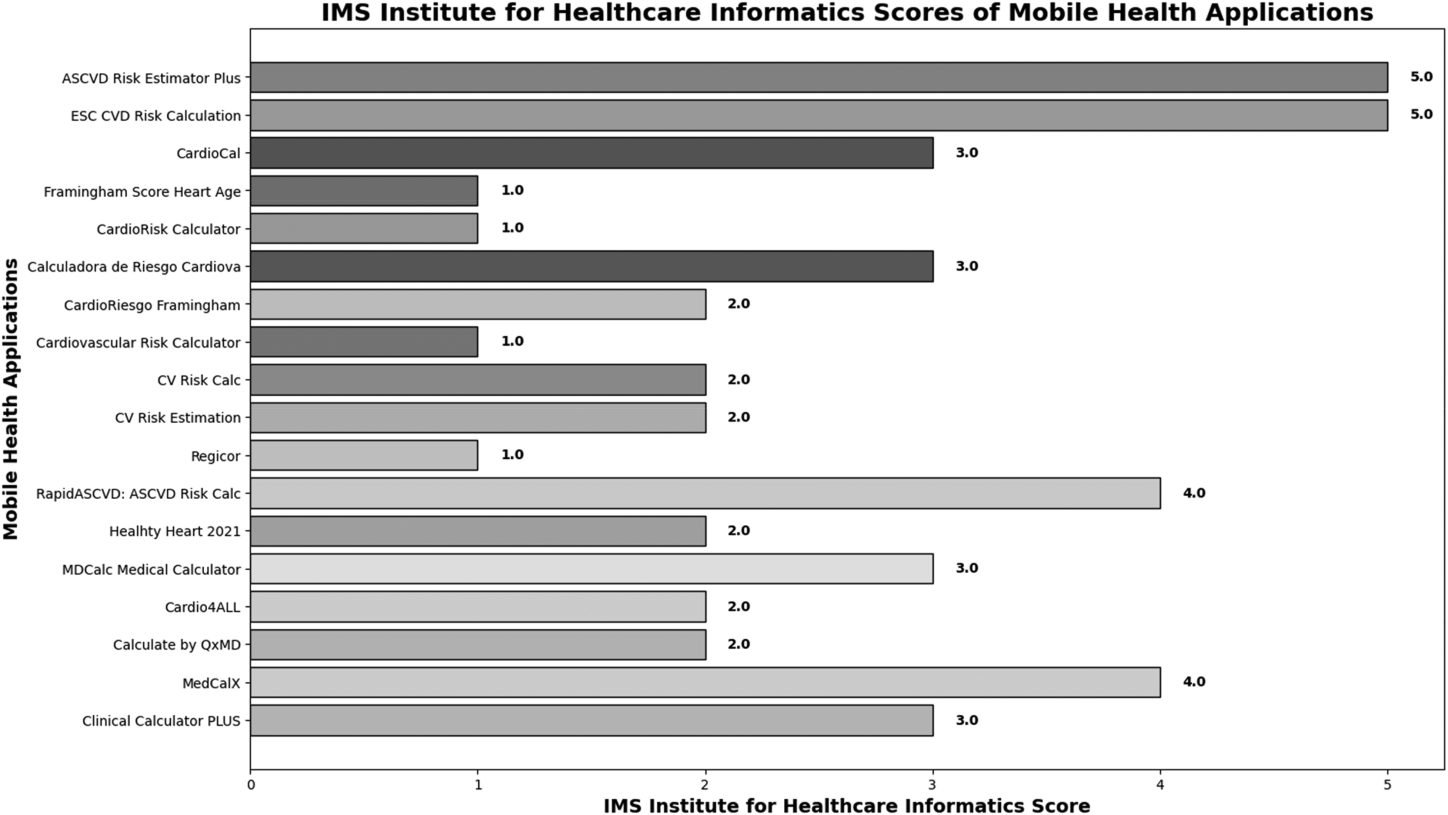
IMS institute for healthcare informatics scores of Mobile health applications.

In IMS Institute for Healthcare Informatics, 4 MHA had high functionality ASCVD Risk Estimator Plus (5 points) ESC CVD Risk calculation (5 points) MDCalc Medical Calculator (4 points), and Calculate by QxMD (4 points). (Figure 4) (further details, see Table 1).

## 4 Discussion

### 4.1 Main findings

In our systematic review of cardiovascular risk assessment apps, our analysis of 35 MHAs available on Apple iOS, Android, and Microsoft store platforms revealed some notable findings. Approximately half of these apps did not align with a validated cardiovascular risk scoring system or were not designed for use by healthcare professionals. Furthermore, only one-third of the evaluated apps achieved overall ratings of “good” or “excellent” in quality assessment for healthcare professionals.

Employing the validated MARS scoring system, only six apps achieved an overall score of 4 or greater (18). These top-performing apps included “ASCVD Risk Estimator Plus” by the American College of Cardiology, “ESC CVD Risk Calculation” by the European Society of Cardiology, “CV Risk Estimation” by United Health Services, Inc., “MDCalc Medical Calculator” by MD Aware LLC, “Calculate by QxMD” by WebMD Health Corporation, “Clinical Calculator PLUS” by Skyscape Medpresso Ins. Among these, the first two MHAs received the highest scores for functionality assessment, both according to the IMS, which evaluates the availability of the functionality, and the MARS score, which measures the quality of app performance (16, 19).

It is important to note that these assessments were conducted as of August 2023, and the MHAs landscape are dynamic. Consequently, while “ASCVD Risk Estimator Plus” and “ESC CVD Risk Calculation” stood out as top performers during our analysis, the landscape may have evolved since our evaluation.

To our knowledge, there has been no prior study evaluating the quality of cardiovascular risk assessment MHAs for use in healthcare professional training and practice.

### 4.2 Comparison between apps

#### 4.2.1 Comparison of risk estimation guidelines

When confronted with the choice between “ASCVD Risk Estimator Plus” and “ESC CVD Risk Calculation”, our top-performing apps, it is essential to consider the foundational guidelines they rely on for risk estimation and recommendations. Notably, the former follows advice from the 2019 Primary Prevention Guideline, 2018 Cholesterol Guideline, and 2017 High Blood Pressure Guideline, while the latter follows advice from the 2019 ESC/EAS Guidelines for the management of dyslipidemias, 2021 ESC Guidelines on Cardiovascular Disease Prevention in clinical practice, and 2018 ESC/ESH Clinical Practice Guidelines for the Management of Arterial Hypertension guidelines (12, 20–24).

#### 4.2.2 ASCVD risk estimator plus app

The “ASCVD Risk Estimator Plus” predominantly focuses on the primary prevention of CVD and provides an adaptation of the original ASCVD Risk Estimator. However, it lacks age-specific risk thresholds for individuals aged 40–75 and employs relatively higher risk percentages to define intermediate and high-risk categories (25). However, it distinguishes itself through its adaptability, supporting the utilization of other risk calculators like the Pooled Cohort Equation (PCE) for lifetime risk calculation by age, sex, and ethnicity, making it suitable for a diverse demographic group, including Asian and Hispanic individuals (26). Furthermore, based on the guidelines, it advises the use of additional biomarkers such as C-reactive protein, apolipoprotein B, lipoprotein (a), and triglycerides for further risk classification for clinician-patient risk discussion (CPRD). Another noteworthy feature is its capability to provide project risk reduction scenarios founded on lifestyle modifications and pharmacological management. Most significantly, it provides each piece of advice with a level of evidence, allowing healthcare providers to make well-informed decisions. Additionally, this app offers the convenience of printing or emailing treatment advice, streamlining communication between healthcare providers and patients, thereby enhancing shared decision-making and CPRD.

#### 4.2.3 ESC CVD risk calculation app

On the other hand, the “ESC CVD Risk Calculation” reflects the comprehensive 2021 ESC Guideline, encompassing both primary and secondary prevention of CVD. This app extends its prevention recommendations to include broader population-level threats to cardiovascular health, such as environmental factors like air and noise pollution and urban planning. Notably, it incorporates the European Systemic COronary Risk Estimation 2 (SCORE2) and SCORE2-Older Persons (SCORE2-OP) risk calculators, a critical feature encompassing both fatal and nonfatal CVD outcomes, enabling precise 10-year atherosclerotic CVD (ASCVD) risk estimation for patients below and over 70 years old respectively (13, 14). However, it is essential to consider that it requires the selection of a specific European region, limiting its specificity for users outside these regions. Furthermore, it does not endorse imaging for further risk stratification or provide an option to determine the therapy impact nor offer specific advice for shared decision-making or CPRD.

#### 4.2.4 Common features and shared decision-making support

In common, both apps share the use of lifetime risk calculators [PCE and LIFEtimeperspective CVD (LIFE-CVD)] to facilitate and foster informed and shared decision-making discussions concerning specific risk factors, such as diabetes mellitus and previous CVD events (26, 27).

#### 4.2.5 Alternative: MDCalc medical calculator app

In addition to these top-performing apps, “MDCalc Medical Calculator” stood out as a noteworthy alternative. This versatile app encompasses a wide array of calculators for various diseases. For cardiovascular risk assessment, it considers the ASCVD risk score based on the 2013 ACC/AHA Guideline on the Assessment of Cardiovascular Risk and the Framingham risk score (26, 27). It provides advice and evidence regarding statin use for the ASCVD score and additional blood pressure advice for the Framingham score. However, it's important to note that the recommendations are based on the 2013 ACC/AHA guidelines and do not include further assessment for risk stratification or features for shared decision-making.

### 4.3 Clinical implications

Quantitative absolute risk assessment has assumed a prominent role in U.S. and international guidelines, facilitating decision-making in primary prevention (28). The choice between “ASCVD Risk Estimator Plus” and “ESC CVD Risk Calculation” should be dictated by the specific preferences and requirements of MHAs users, supported by guideline recommendations and individualized patient needs. Should users prioritize a comprehensive approach encompassing both primary and secondary prevention, age stratification, alongside the consideration of broader environmental factors, “ESC CVD Risk Calculation” may emerge as the preferred choice. However, “ASCVD Risk Estimator Plus” could prove to be the more appropriate selection if users demand greater flexibility in risk calculation, biomarker or imaging consideration, and comprehensive team-based care for risk factor management and shared decision-making. It is also worth mentioning that users should consider their ethnicity, as both guidelines provide specific multipliers for select populations, thereby ensuring a personalized approach to risk assessment.

Despite “MDCalc Medical Calculator” being one of the top-performing apps, several implications need to be considered. Users may find it valuable as it contains a wide variety of scores for different subspecialties, making it a practical app for daily clinical decision-making (29). When contemplating a primary prevention approach, practitioners can choose to use the 2013 ACC/AHA Guideline on the Assessment of Cardiovascular Risk or the Framingham risk score (intended for use in non-diabetic patients aged 30–79 years with no prior history of coronary heart disease or intermittent claudication) (26, 27). For secondary prevention, the app offers the ASCVD Risk Algorithm including Known ASCVD from AHA/ACC (30). This app provides advice and evidence for each risk assessment tool included however, the recommendations are based on the 2013 ACC/AHA guidelines and do not include further assessment for risk stratification of features for shared decision-making.

Language also poses a consideration. The top three performing apps are exclusively available in English, creating a language barrier for non-English speaking regions. Conversely, “Calculate by QxMD”, another high-performing app, offers a variety of languages and, similar to “MDCalc Medical Calculator”, provides the ASCVD risk score based on the 2013 ACC/AHA Guideline on the Assessment of Cardiovascular Risk or the Framingham risk score (26, 27).

Our findings, in conjunction with related studies that show little evidence-base for commercially available MHAs, highlight that the majority of MHAs are not suitable for healthcare provider use (31, 32). A critical need exists for periodic reviews utilizing validated scoring systems to identify accurate and reliable MHAs for cardiovascular risk stratification. Given the prevalence of smartphones in daily healthcare practice, healthcare professionals increasingly need dependable electronic resources (33). The high-performing apps identified in this study are highly valuable tools for daily cardiovascular risk assessment. Specifically, “ASCVD Risk Estimator Plus” can facilitate shared decision-making and CPRD potentially strengthening clinician-patient relationships, enhancing patient engagement, and promoting medication adherence (34). Further research is necessary to assess healthcare professionals’ specific needs and develop interactive MHAs that optimize global risk scores, enhancing adherence to guideline-based therapy (35). Finally, research should focus on evaluating the impact of MHAs on clinical practice and patient outcomes.

### 4.4 Limitations

The strengths of this study include the utilization of a widely accepted, validated system for standardized analysis of MHAs. Furthermore, we conducted an extensive search encompassing both paid and unpaid apps available on both Apple iOS and Android platforms. However, it is essential to acknowledge certain limitations including the rapid pace of product development, availability, and updates which present challenges in timely evaluation. There is a need for guidelines assessing MHAs in quality and functionality for healthcare professional use. Moreover, our screening and evaluation process was not conducted by cardiologists or cardiology fellows but rather by medical students trained in systematic reviews at Red de Eficacia Clínica y Sanitaria (REDECS). Finally, we did not assess cardiovascular risk apps incorporating the Multi-Ethnic Study of Atherosclerosis (MESA) 10-Year CHD Risk with Coronary Artery Calcification due to its limited availability in only one app, which was developed by the same study and limited our comparison between the other risk calculators (36). However, it can be considered as a complementary tool for risk stratification in patients with borderline or intermediate risk (20).

## 5 Conclusions

A significant gap is evident in the availability of high-quality MHAs designed for healthcare professionals specifically designed for healthcare professionals to facilitate shared decision-making in cardiovascular risk assessment. This gap underscores the need for efforts towards the development of comprehensive guidelines aimed at evaluating the quality and functionality of MHAs intended for provider use, moving beyond the scope of self-care management apps. Additionally, continuous app updates and enhancements are essential to ensure healthcare professionals have access to language-diverse and up-to-date tools for effective risk assessment and management. Lastly, while the selection of MHAs should be guided by individual preferences, it is essential that they align with the most current clinical practice guidelines, thereby emphasizing the importance of evidence-based decision-making in optimizing patient outcomes.

## Data Availability

The original contributions presented in the study are included in the article/Supplementary Material, further inquiries can be directed to the corresponding author.

## Appendix

Summary and reflections box.

**Table:**

Summary
• Main findings: our review of 35 cardiovascular risk apps showed that many apps are not aligned with validated risk scores or are not meant for healthcare professionals. Only one-third received high quality ratings. Top apps include “ASCVD Risk Estimator Plus” and “ESC CVD Risk Calculation,” noted for their high functionality and quality. The app landscape changes rapidly, so these findings may evolve. • App comparison: “ASCVD Risk Estimator Plus” and “ESC CVD Risk Calculation” are the top apps, with “ASCVD” offering flexibility and detailed risk scenarios, while “ESC” includes environmental factors and European risk calculators. “MDCalc Medical Calculator” is useful but lacks some features for shared decision-making. • Clinical implications: choose apps based on specific needs, like comprehensive prevention or flexible risk calculation. Language support and guideline alignment are important
Reflections
• Clinical relevance: high-performing apps like “ASCVD Risk Estimator Plus” can improve patient engagement and decision-making. There's a need for more high-quality, language-diverse apps. • Limitations: we faced challenges like rapid app updates and evaluation by non-specialists. Future research should address these gaps and assess how apps impact clinical practice. • Future directions: we need updated guidelines for app quality and functionality. Future work should focus on creating interactive apps and assessing their real-world impact
