# Supplementary material for: Mobile health apps for cardiovascular risk assessment: a systematic review
Source: Front Cardiovasc Med. 2024 Sep 23;11:1420274. doi: 10.3389/fcvm.2024.1420274 (PMC11456540; doi:10.3389/fcvm.2024.1420274)
Supplement: Supplementary file 1 [file Table1.pdf]

SUPPLEMENTARY MATERIAL

Table Supplementary 1. Reason of excluded mhealth apps.

App name	Platform	Developer	Reason for exclusion
CardioVisual: Heart Health	IOS/Android	MedicalVisual, Inc	Did not open
Riesgo Cardiovascular App	Android	Niriom	Did not open
Test del Corazón – calculadora del riesgo	IOS	Pears Health Cyber s.r.o.	Did not comply with a risk scoring system
CURRENT Dx Tx Cardiology	IOS/Android	Skyscape Medpresso Inc	It did not measure cardiovascular risk. Educational purpose
Riesgo Cardiovascular UMariana	Android	Niriom	Did not open, required an institutional account
Goldman Cardiac Risk	IOS	DOCTOT	Uses "Goldman Cardiac Risk"
Test del Corazón-calculadora del riesgo	IOS	Pears Health Cyber s.r.o.	Did not comply with a validated risk scoring system
My Heart Risk	IOS/Android	Creighton University	Did not open
EPI.RxISK	IOS	University of Alberta	Uses "Alberta Vascular Risk Reduction Community Project"
DM2 360°	IOS	Boehringer Ingelheim Pharma GmbH & Co.KG	Uses a different score not specified
Lipid manager	IOS/Android	American College of Cardiology Foundation	Duplicated
NLA ASCVD Risk Assessment Tool	IOS	The National Lipid Association	It did not measure cardiovascular risk. Educational purpose
